# HYBRID: Ambulatory Robotic Gait Trainer with Movement Induction and Partial Weight Support

**DOI:** 10.3390/s19214773

**Published:** 2019-11-02

**Authors:** Eloy Urendes, Guillermo Asín-Prieto, Ramón Ceres, Rodrigo García-Carmona, Rafael Raya, José L. Pons

**Affiliations:** 1 Department of Information Systems Engineering, University San Pablo CEU, Boadilla del Monte, 28688 Madrid, Spain; 2 Neural Rehabilitation Group, Cajal Institute, CSIC—Spanish National Research Council, 28002 Madrid, Spain; 3 Legs & Walking AbilityLab, Shirley Ryan AbilityLab, Chicago, IL 60611, USA; 4 Department Biomedical Engineering & Department Mechanical Engineering, McCormick School of Engineering, Northwestern University, Evanston, IL 60208, USA; 5 Department of PM&R, Feinberg School of Medicine, Northwestern University, Chicago, IL 60611, USA

**Keywords:** gait, trainer, partial weight suspension, induction of movements, disability, exoskeleton, lower body rehabilitation, robotic rehabilitation

## Abstract

Robotic exoskeletons that induce leg movement have proven effective for lower body rehabilitation, but current solutions offer limited gait patterns, lack stabilization, and do not properly stimulate the proprioceptive and balance systems (since the patient remains in place). Partial body weight support (PBWS) systems unload part of the patient’s body weight during rehabilitation, improving the locomotive capabilities and minimizing the muscular effort. HYBRID is a complete system that combines a 6DoF lower body exoskeleton (H1) with a PBWS system (REMOVI) to produce a solution apt for clinical practice that offers improves on existing devices, moves with the patient, offers a gait cycle extracted from the kinematic analysis of healthy users, records the session data, and can easily transfer the patient from a wheelchair to standing position. This system was developed with input from therapists, and its response times have been measured to ensure it works swiftly and without a perceptible delay.

## 1. Introduction

Assisted gait training and rehabilitation have a high impact on healthcare and are characterized by scientific and technical challenges [1,2,3]. Recent research in this field has translated into several devices, ranging from prostheses and orthoses to walkers and gait trainers, that provide users with aids designed to help with particular disabilities.

There is no consensus on which program is best suited for gait rehabilitation or which tools are most useful, especially for spinal cord injury (SCI) [4,5]. However, repetitive movement strategies have proliferated in clinical practice [6,7]. They are designed to stimulate the central pattern generators (CPGs), responsible for generating coordinated movements [5,8]. This manual-therapy-based training is held back by lack of personnel and the physical effort it demands from the therapist, severely limiting its applicability. Robotics-based rehabilitation offers a unique opportunity to solve this problem, improving training intensity and quality. By assisting both the therapists and the patients in performing a rhythmic and synchronized lower body movement, robotic devices have the potential to greatly reduce the physical effort needed, and thus decrease the injuries of therapists derived from this effort.

## 2. Related Work

Wearable exoskeletons that can induce musculoskeletal movement, and therefore help the user perform a healthy gait pattern, are a very popular way of using robotic devices for rehabilitation. Most of them, like Ekso Bionics [9], HAL [10], Vanderbilt exoskeleton [11], or ReWalk [12] are 4DoF (degrees of freedom) lower-limb exoskeletons, and actuate both the hip and knee on the sagittal plane. Recent studies [13,14] have demonstrated that training using HAL is effective in improving ambulatory mobility for patients with stroke or spinal cord injury. Robotic training led to an improvement in walking parameters and in balance abilities, with increases in the WISCI-II score. Similar results have been achieved using Ekso Bionics [15]. Other devices such as MindWalker [16], CUHK-EXO [17], or XoR2 [18] include hip rotation. These ambulatory exoskeletons need crutches to provide stabilization and control the lateral balance during movement. This setup is infeasible for patients that lack good upper body strength or biomechanical coordination. On top of that, they introduce the risk of falls and produce fatigue. These factors make such exoskeletons impractical for many users.

This problem could be avoided by using PBWS (partial body weight support) solutions that unload part of the patient’s body weight by suspending them. This way, the percentage of body weight that the lower body must support during rehabilitation therapy can be tightly controlled. Such strategies have the potential to improve the locomotive capabilities of people with motor deficit by minimizing the muscular effort required and simultaneously reducing the forces and pressures exerted over the user’s skeletal system [19]. Several studies have proved the huge benefits that PBWS solutions have on clinical practice [20,21].

However, PBWS was traditionally implemented using fixed structures with either treadmill or rigid frames. Recent solutions have seen the introduction of robotic gait trainers that can combine assisted leg movement with partial weight suspension. This way, crutches are not needed and falls can be totally avoided. These two features are considered mandatory for patients with systemic motor deficit.

Devices like Lokomat [22], GaitTrainer [23] LOPES [24], ALEX [25] or C-ALEX [26] integrate an exoskeleton for particular body regions with treadmill and PBWS systems. These solutions enable reproducible and intense therapy sessions while logging objective measures to assess the exercise’s results. These devices are limited in the sense that the patient remains in place, and therefore their proprioception, posture, and dynamic balance systems are not being properly trained and receive conflicting stimuli.

To address these shortcomings, new ambulatory training systems which allow a higher freedom of movement from the patient have been designed, such as WalkTrainer [27] Nature-gaits [28], SUBAR [29], EXPOS [30], the device presented in [31], MLLRE [32], or MOPASS [33]. However, solutions such as these present several drawbacks, like the inability to easily move the patients from their wheelchairs to standing position. On top of that, these trainers cannot generate a gait movement synchronized with the system’s absolute speed or force the patient to maintain a posture conductive to put on an exoskeleton—a process involving important physical effort and lengthy preparation times.

This work tries to bridge this gap by presenting an innovative self-propelled ambulatory system for gait rehabilitation with lower body movement induction, PBWS and body stabilization designed for clinical environments, named HYBRID (Hybrid Technological Platform for Rehabilitation, Functional Compensation and Training of Gait in SCI Patients). Our solution enables practitioners to easily transfer patients from a wheelchair to standing position, suspend their weight, and provide assisted gait patterns extracted from healthy users, all while offering safety and comfort. This system is easy to use for both users and practitioners.

The developed system has been financed by the Spanish *Plan Nacional*, with support from interested partners like the *Hospital Nacional de Parapléjicos de Toledo* (HNPT) and the *Instituto Nacional de Educación Física* (INEF), and has received input from other experts in the fields of rehabilitation and gait analysis, helping to focus on the more relevant design issues.

## 3. Materials and Methods

**Figure 1:**
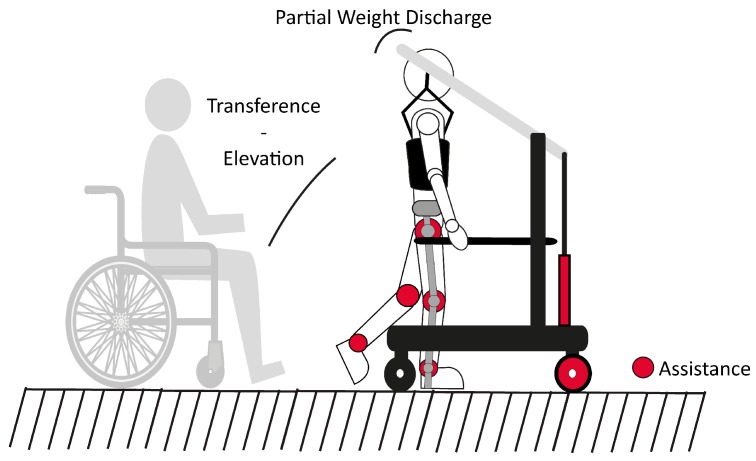
General view of the HYBRID device.

The HYBRID device is a gait trainer based on a double support system (Figure 1): a bilateral 3DoF per leg (6DoF total) lower-limb exoskeleton (H1 [34,35,36]) and a PBWS system (REMOVI). The former is an exoskeleton that can perform a prerecorded walking pattern while registering the angular positions of its six actuated joints (ankles, knees, and hips). The latter is an active PBWS system that supports both the exoskeleton and the user through a harness. For the sake of clarity, this section is divided in three parts, with each of the first two devoted to a different subsystem of HYBRID: H1 and REMOVI. Each part is further divided into several smaller subsections that detail the mechanical structure and electromechanical elements, sensors, and software platform of the H1 and REMOVI modules. Finally, the last part details how the communication between the exoskeleton, the PBWS, and the therapist’s computer is achieved.

The H1 lower-limb robotic exoskeleton was created by the Neural Rehabilitation Group (NRG) from the Cajal Institute of CSIC as part of the HYPER project [37]. It is built upon the experience acquired in previous projects [38,39,40,41] and is under active development [42,43]. The H1 is a medical device designed for the clinical environment, to provide training and rehabilitation of gait over ground for patients suffering from several pathologies, such as stroke, spinal cord injury, or cerebral palsy. It can support cadences of up to 0.5 m/s (1.8 km/h) and produce a gait pattern extracted from the kinematic analysis of healthy users. This exoskeleton can also be locked in a forced standing position, stabilizing the patient by blocking the motors.

The REMOVI PBWS system is a new development designed to reduce the physical effort required by both the patient and the practitioner to a minimum. Toward this aim, it needs to be self-propelled, provide a transfer operation that can move the patient from a wheelchair to standing position (and vice-versa), allow the user to freely walk around with partial weight suspension, and interface and integrate with the H1 exoskeleton module.

### 3.1. H1 Exoskeleton

#### 3.1.1. Mechanical Structure and Electromechanical Elements

Since gait happens mainly in the sagittal plane, the exoskeleton has been designed to actuate over it. Unlike most exoskeletons (commercially available or in the literature), it also has powered ankles. This feature is especially important since the ankle bears high torques during gait [44], is responsible for providing balance and attaining the standing position [45,46], and supports considerable body weight [46]. Additionally, heel contact, toe off, and plantar sensitivity play a very important role in balance proprioception [47], and a proper ankle movement is needed to produce these sensations.

**Figure 2:**
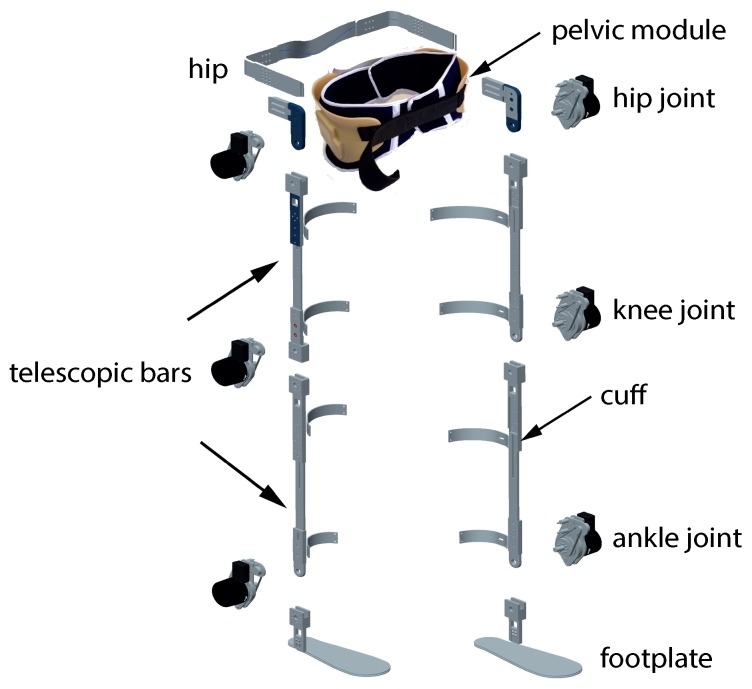
Exploded view of the H1 exoskeleton mechanical structure.

**Figure 3:**
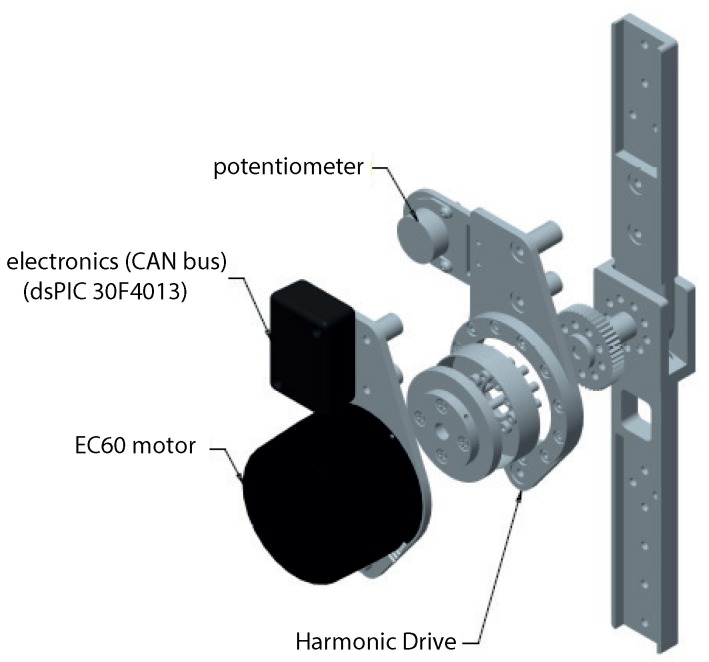
Exploded view of a joint actuation module. CAN: Controller Area Network.

The H1 exoskeleton is comprised of two (one per leg) mechanically independent 3DoF lower-limb orthoses, actuated at the hip, knee, and ankle joints in the sagittal plane. Figure 2 shows that each orthosis is made up of four rigid telescopic bars that correspond to the pelvis, femur, tibia, and foot. They are connected by actuation modules (see Figure 3) that match the location of human joints. Both orthoses are connected through a rigid hip structure and a deformable pelvic module made out of polymer, to better adapt to the user’s waist and abdominal contour. This setup allows for small adduction and abduction non-actuated hip movements. The exoskeleton is adjustable in width, depth, and height to match the user’s characteristics (1.50 m to 1.90 m height, up to 100 kg weight), covering more than 95% of the target population [48].

The exoskeleton’s range of motion (ROM) corresponds to that of a healthy individual [49] and can be seen in Table 1. This ROM allows users to also perform sit-to-stand and stand-to-sit motions. The H1 design and materials selection has taken into account ergonomics and comfort issues.

Attachment to user’s body is achieved via two elements: the pelvic module for the hip, and the cuffs for the leg segments and feet. Both are foamed and fitted with velcro straps. Most of the structure is made of aluminum due to its resistance and low weight: the exoskeleton weights only 13 kg, and its battery adds another 2 kg.

Each orthosis includes three brushless direct current (BLDC) motors (i.e., one for each of the joints), coupled to the axis of the joint. The BLDC motor used for all the joints is a Maxon EC60-100W-24V, 60 mm diameter, nominal speed 4250 rpm, and nominal torque 227 mN·m. The coupling to the output link is done via a Harmonic Drive gearbox: model CSD20-160-2AGR, with a reduction ratio of 160:1. This setup leads to a nominal speed of 26.5 rpm, constant torque of 35 N·m, and peak torque of 180 N·m, providing a high torque and low speed, both suitable to generate the movement on each of the joints [50]. All motors are driven by Advantech AZBH12A8-24 V power drivers, connected to the exoskeleton’s main controller through an analog channel used for pulse width modulation (PWM).

#### 3.1.2. Sensors

The H1 exoskeleton is equipped with kinematic and force sensors [51]. Concerning the former, each joint is equipped with a 10 kΩ high-precision one-turn potentiometer, by Vishay Spectrol, model 157S103MX, with high linearity and long rotational life. This potentiometer is used as the joint angular sensor. It is directly coupled to the output axis via a transmission belt, providing a direct measure of the joint angle.

The footplates are fitted with two force-sensing resistors (FSRs) each (Interlink Electronics 406) placed under the user’s heels and toes. They allow the detection of four gait events (i.e., initial contact, flat foot, heel off, and toe off), enabling the identification of the different phases of gait.

Finally, the exoskeleton as a whole has an ON/OFF switch and each orthosis has a button that the user can press to perform a step with that leg when the step-by-step mode is activated.

**Figure 4:**
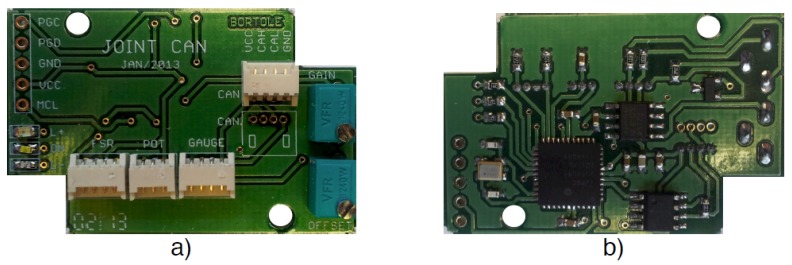
Custom-made boards for the acquisition and transmission of sensor data. (**a**): Top side and (**b**): Bottom side of the boards.

The sensory data are acquired by a custom-made board (see Figure 4), one for each joint, that performs the signal conditioning, acquisition, and digitalization functions and sends the processed data to the main controller through a CAN (Controller Area Network) bus. This board’s microcontroller is a dsPIC30F4013 by Microchip. The FSR data from the footplates is processed by the ankle boards.

#### 3.1.3. Computing Platform and Software

The main controller of the H1 exoskeleton has been programmed to run in a real-time environment on a PC/104 computer. The PC/104 main board module is connected to two Diamond Systems Corporation DMM-32X-AT acquisition boards, with 32 analog input channels (16 bits each, unused), and 4 analog output channels (8 bits each, used to drive 6 motors); *a* UDP communication board (Advantech PCM-363); a CAN communication board (C2-104); and an Advantech PCM-3910 power supply module.

**Figure 5:**
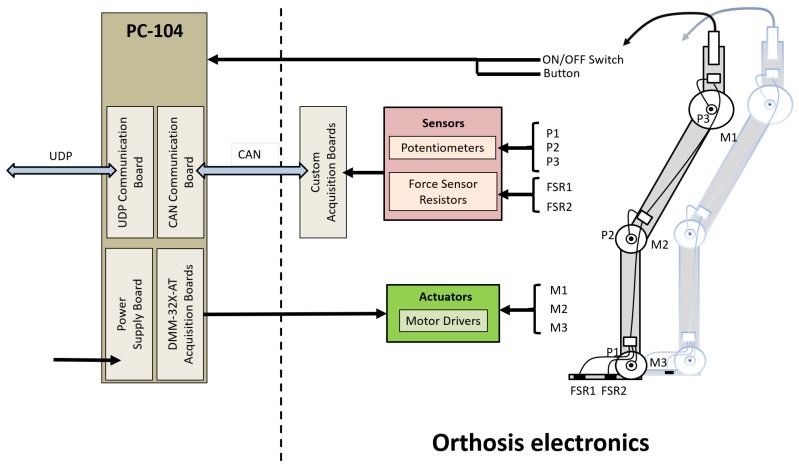
H1 exoskeleton electronics architecture. UDP: User Datagram Protocol.

Figure 5 shows how the sensors, actuators (motors), and electronics (both custom-made and stock boards) fit together to enable the movement of each leg. The PC/104 computer is shared by both orthoses. The UDP module is used to connect the exoskeleton to the REMOVI PBWS subsystem and a PC that the practitioner can use to control and monitor the therapy session.

**Figure 6:**
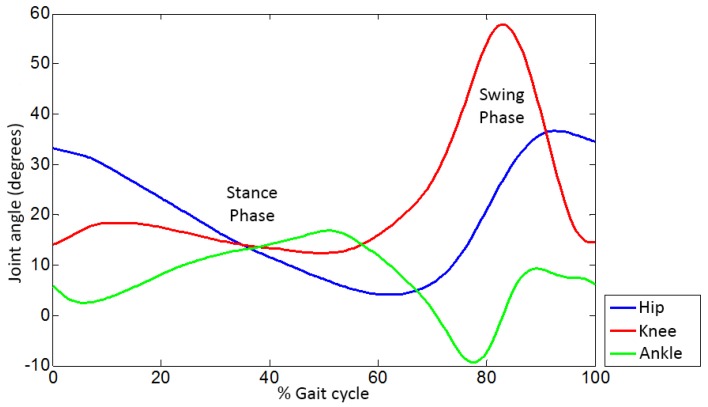
Ankle, knee and hip trajectories for the gait pattern.

All the exoskeleton software was implemented using Simulink. The trajectories designed for the H1 match a normalized gait pattern, provided by the *Departamento de Salud y Rendimiento Humano* of INEF by measuring 29 healthy female and 33 healthy male individuals walking slowly (around 0.25 m/s) using a VICON photogrammetry system. The resulting gait is depicted in Figure 6, with trajectories for ankle, knee, and hip.

**Figure 7:**
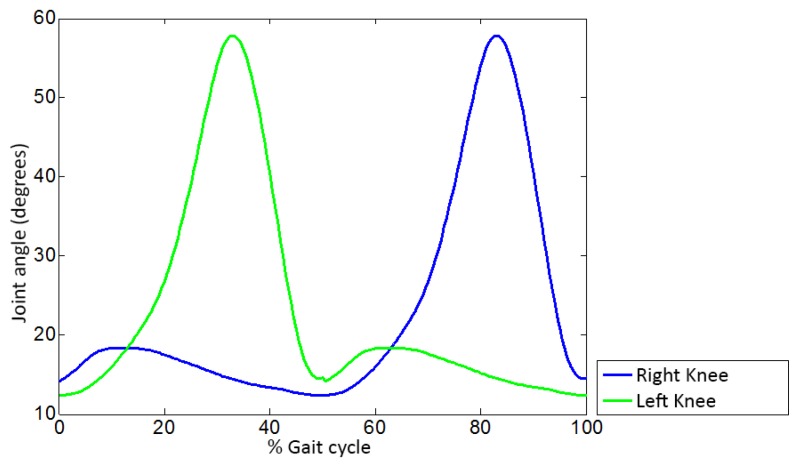
Gait pattern offset.

These trajectories assume symmetry between both legs and are stored in tables used by a pattern generator module to produce a gait cycle. To implement a continuous gait they play cyclically and simultaneously for both legs, with a 50% offset between them—the normal behavior for a healthy gait [45]. This offset is shown in Figure 7, which depicts only the knee joint for the sake of clarity.

The pattern generator module supports two modes: step-by-step and continuous gait. In both of them the exoskeleton can change its speed from 0.12 to 0.28 m/s.

In the step-by-step mode, the user can perform each step independently by pressing the corresponding button. Each press induces a gentle oscillation movement in the advancing leg that translates into a single step, while the other limb moves slightly to ensure stability. Both movements stop when the advancing leg finally reaches the ground plane. This way, the patient can control pace themselves.

In the continuous gait mode, the gait pace is set and maintained. The movement is not stopped, with both legs performing the full cycle without stops between steps.

When gait is not induced (start, stop, and end of exercise) the H1 exoskeleton assumes a forced standing position, with hip, knee, and ankle at $0^{{}^{\circ}}$.

We implemented a finite-state machine to change between modes with five possible states (lock, left, right, left-stop, and right-stop). The output of this machine tells the pattern generator the selected mode. This translates to the following conditions:

- Always starts at the lock state; after the user has fitted the exoskeleton and activated it, the H1 stays in the forced standing position. This avoids a fall due to an involuntary movement by the patient.
- If the continuous gait mode is selected, the session starts with the right leg (to improve the learning curve of the system). This mode uses the left and right states that, when strung together, create a continuous pattern.
- If the step-by-step mode is selected, the patient can choose which leg to start with. This mode uses the left-stop and right-stop states, which generate a split pattern.
- Modes can be changed without first reaching the lock state. The system waits until the current state is finished, and then switches to a state appropriate to the new mode (continuous gait or step-by-step).
- To move out of the left-stop and right-stop states, the patient must press one of the buttons.

To avoid sudden changes in joint positions while shifting modes (lock, continuous, and step-by-step), the maximum difference between joint angular references is set to $3^{{}^{\circ}}$, which translates into a gentle and controlled movement.

Modes are selected by the practitioner using a GUI (graphical user interface) running on a computer connected via WiFi or Ethernet to the exoskeleton. This connection and user interface are detailed later.

The software stack for the H1 exoskeleton is split into:

- A high-level layer responsible for determining the movement type, defining the joints’ trajectories, synchronizing the three joints of each leg, and coordinating the combined motion of both limbs. This layer uses the stored pattern and implements the previously mentioned state machine.
- A low-level controller that maps the trajectories defined by the high-level layer to specific joint positions, implemented with a PID (Proportional Integrative Derivative) controller.

### 3.2. REMOVI PBWS

#### 3.2.1. Mechanical Structure and Electromechanical Elements

The REMOVI PBWS must support patients of similar size and weight to those allowed by the H1 exoskeleton: 1.60 m to 1.90 m height and up to 120 kg weight (including the weight of both the patient and the exoskeleton). Again, these parameters cover more than 95% of the target population [48]. In this case the height is especially important for the design, since the arms’ final positions must leave a clear walking area in front of the patient. Additionally, the wheelchair-to-standing position transfer process needs to be safe and progressive in order for the users not to feel that they are under risk of falling before or during this process. The proposed mechanism has a gentle upwards trajectory that is nonetheless fast enough to avoid anxiety due to the feeling of instability while being raised.

**Figure 8:**
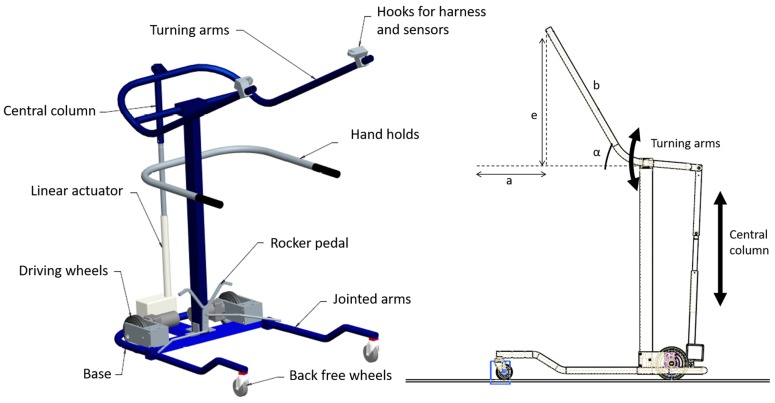
REMOVI mechanical structure.

To maximize the feeling of safety provided by REMOVI, this module uses a built-in harness that spans from thorax to perineum and bears the weight of both the user and the H1 exoskeleton. This harness is suspended using a symmetric mechanism in the transverse plane (Figure 8). This setup increases the stability and balance of the patient’s upper body and favors the weight transition from one leg to the other during gait. The REMOVI module also has a wheeled base with two frontal driving wheels, actuated by two direct current (DC) 24 V motors and two rear free wheels. A central column with two turning arms is in charge of the transference from the wheelchair to the standing position and vice versa. These supporting arms are actuated by a 24 V DC linear actuator and also perform the partial body weight support function. There are two hooks at the end of these arms to which the harness and force sensors are fixed.

The turning arms’ trajectories produce two combined movements: elevation (e) and approach (a). Both are shown in Figure 8, and the relationship between them is characterized as follows (where b is the arm’s length):

(1) e=b·sinα,

(2) a=b·(1−cosα).

**Figure 9:**
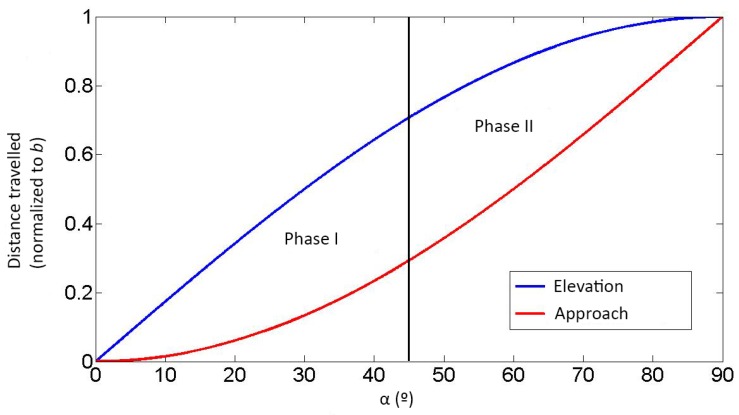
REMOVI mechanical structure.

The wheelchair-to-standing transference can be characterized as two different phases, depicted in Figure 9. Phase I is dominated by elevation, while the opposite happens in phase II, where the approach component grows more quickly. This two-phase transference was designed to reduce the risk of falling and avoid the wheelchair being dragged.

For the harness, after a careful study of several solutions, we selected a thorax and groin Biodex model. Its ergonomics and, more importantly, its capacity to properly distribute weight were the deciding factors. This last feature can be used to avoid having high-pressure points that could disturb the user or even produce sores. This harness can be adjusted to different sizes and thorax perimeters, covering the desired target population.

For this transference process and the weight support modulation we chose a Linak LA345100+0L300041 24 V actuator that can push up to 5000 N, pull up to 4000 N, and has a maximum lifting speed of 10 mm/s with a stroke length of 300 mm. To measure the arms’ angular positions, a potentiometer is affixed to the axis that joins them.

To be capable of self-propulsion, the REMOVI is equipped with two DC Kelvin K80-63.105 24 V tractor motors with a reduction ratio of 53.3:1 and a torque factor of 38.9. The nominal torque is 6.61 N·m, the speed is 69 rpm, and the power 50 W. Put together, they are strong enough to move both platform and user. Each motor’s axis is coupled to an HEDS-5540A11 encoder with a resolution of 500 pulses per lap, tasked with measuring the PBWS speed so it can be controlled.

#### 3.2.2. Sensors

The hooks on the ends of the REMOVI arms also contain two AMTI FS6-500 triaxial force sensors. These sensors can measure force in the *X*, *Y*, and *Z* axes: up to 2200 N for the *X* axis, and up to 1100 N for the *Y* and *Z* axes.These thresholds are sufficient for an expected maximum user weight of 100 kg. The data retrieved by these sensors (*X*, *Y*, and *Z*) are used to monitor the interaction between the user and the REMOVI PBWS during the elevation process and evaluate the swinging produced during the displacement of the whole system once the user is supported. The arms’ angular positions (α) are measured with a potentiometer affixed to the axis that connects them.

**Figure 10:**
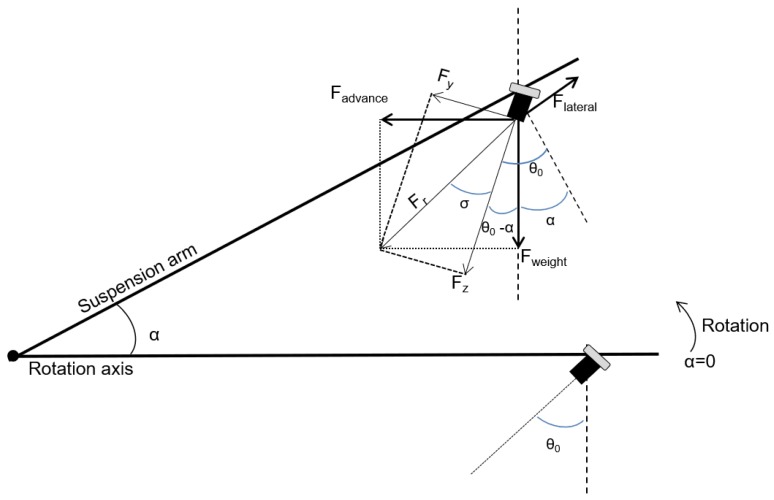
Force component extraction from the sensor measurements.

Figure 10 shows how the relevant force components are extracted, using the following equations:

(3) $$F_{weight}=F_{r}\cdot \cos\left(\sigma +\theta_{0}-\alpha \right),$$

(4) $$F_{advance}=F_{r}\cdot \sin\left(\sigma +\theta_{0}-\alpha \right),$$

(5) $$F_{lateral}=F_{x},$$

(6) $$\cos\left(\sigma \right)=F_{z}/F_{r}.$$

The three calculated components are used to determine the weight supported by the structure (to calibrate the partial suspension percentage), the forward advance, and the lateral movements (for balancing purposes). These equations are implemented in a software data acquisition block.

In addition to these forces, in order to properly characterize the interaction between the user (and the H1 they wear) and the REMOVI system, it is paramount to measure the distance between the patient and the platform itself. The advance speeds of the H1 exoskeleton and the REMOVI PBWS can be programmed so that they match each other, but in practice this is not enough to maintain an optimal relative position between both: slightly higher or lower resistance and contribution to movement by the patient, turns, stops, changes in pace and small differences in gait produce errors that add up and must be corrected. Therefore, it is necessary to monitor the real speeds of both modules dynamically, implementing a feedback loop that maintains a proper distance.

To measure this separation we have used an ultrasound UNDK 30U6103/S14 module, with a range between 100 and 700 mm and a 3 mm resolution. This sensor is placed in the uppermost part of the central column, pointing to the user’s waist. The measured analog signal is sent to the on-board computer (another PC/104, different from the one on the H1 exoskeleton) that regulates the REMOVI speed so it acts as a slave subsystem to the exoskeleton (master), adapting the PBWS’s speed to the user’s pace. To measure the PBWS displacement speed, each DC motor’s axis is coupled to an HEDS-5540A11 encoder with a resolution of 500 pulses per lap, whose data are also sent to this PC/104.

Finally, the REMOVI is also equipped with a joystick that provides manual control for the subsystem’s movements, if desired.

#### 3.2.3. Computing Platform and Software

**Figure 11:**
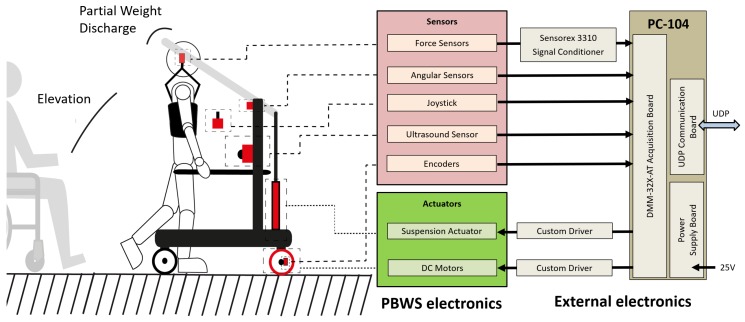
REMOVI electronics architecture. PBWS: partial body weight support.

Like the H1 controller, the REMOVI software runs in a real-time environment on a PC/104 computer and was programmed using Simulink. The PC/104 main board module is connected to a Diamond Systems Corporation DMM-32X-AT acquisition board (whose input channels are connected to all the sensors and three of its four output channels are used to control the motors through two custom drivers); a UDP communication board (Advantech PCM-363); and an Advantech PCM-3910 power supply module. The force sensors are also routed trough a Sensorex 3310 signal conditioner, since they need a stable energy supply and their signals must be amplified and filtered before the PC/104 can process them. This architecture is depicted in Figure 11. Again, the UDP module is used to connect the exoskeleton to the REMOVI PBWS module and the therapist’s computer.

**Figure 12:**
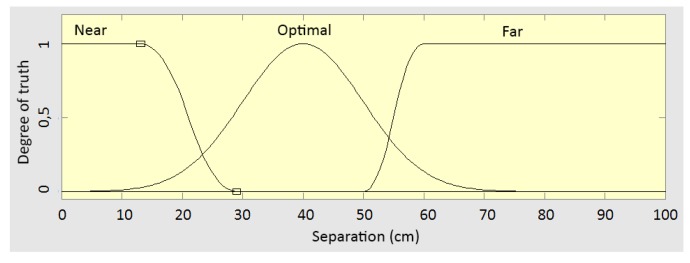
REMOVI distance control input membership functions.

The synchronization between the H1 exoskeleton and the REMOVI PWBS is achieved through a feedback loop (implemented using Matlab’s Fuzzy Logic Toolbox) that controls the REMOVI speed. The input to this loop is the distance retrieved by the ultrasound sensor. The accepted range goes from 0 to 100 cm. We have defined three possible zones (near, optimal, and far distance) including this range and three corresponding input membership functions (Figure 12):

- Near zone: Z-shaped membership function with a=13.1 and b=29.
- Optimal zone: Symmetric Gaussian membership function with σ=40 and μ=10.
- Far zone: S-shaped membership function with a=50 and b=60.

**Figure 13:**
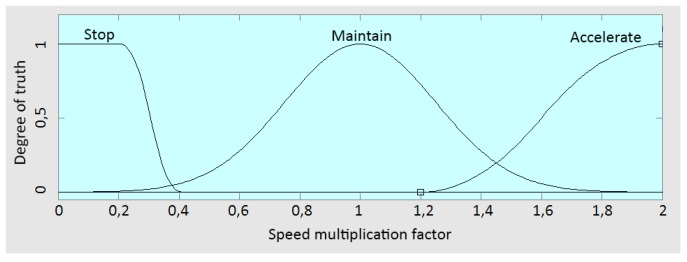
REMOVI distance control output membership functions.

In a similar way, we have defined three output membership functions (Figure 13) that determine three output modes:

- Stop: Z-shaped membership function with a=0.2 and b=0.4. Mapped to the near zone.
- Maintain speed: Symmetric Gaussian membership function with σ=0.25 and μ=1. Mapped to the optimal zone.
- Accelerate: S-shaped membership function with a=1.2 and b=2. Mapped to the far zone.

The outputs of these functions range between 0 and 2. This factor multiplies the H1 exoskeleton speed value and applies it to the REMOVI, with the result of increasing or reducing the distance between both subsystems. The maximum factor of 2 was chosen because REMOVI’s maximum speed is twice the exoskeleton’s.

**Figure 14:**
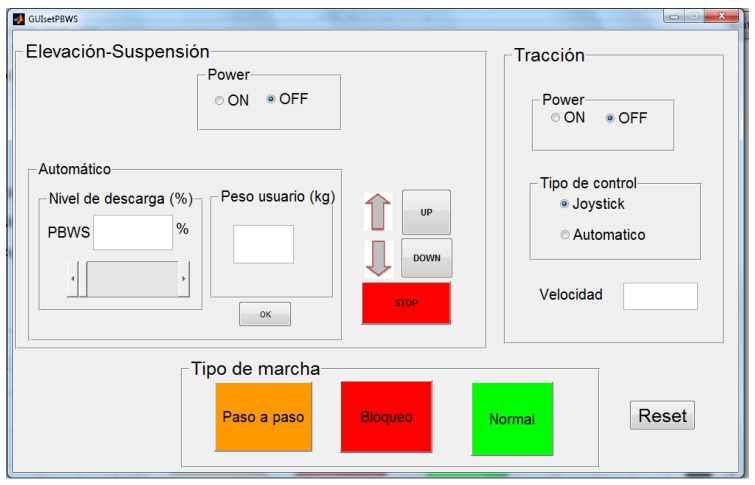
Therapist configuration graphical user interface (GUI).

The elevation process is controlled by the therapist using a graphical interface (Figure 14) with three buttons: up, down, and stop. The provided interface also allows the practitioner to input the desired weight support percentage and movement speed (the same for both the H1 and REMOVI subsystems).

The suspension level can be controlled by the patient and/or the practitioner, both before transferring to standing position and during the session.

The provided software automatically manages the wheelchair-to-standing transition (through arm rotation), level of weight support, and HYBRID speed. This is possible thanks to the data retrieved by the sensors of both the H1 and REMOVI, which allow the system to adapt to the patient’s weight. The forces exerted by the user are also stored for later analysis by the therapist. This way, this information is used not only for weight support control, but also to characterize the patient’s gait.

### 3.3. Subsystems Communication

The H1 exoskeleton, the REMOVI PBWS and a standard computer operated by a therapist comprise a communication network that uses UDP (User Datagram Protocol) as a transport protocol. Using this network, the practitioner’s computer can configure the exoskeleton and PBWS parameters using a graphical user interface (already shown in Figure 14). Both the exoskeleton and the PBWS can be connected using either cabled Ethernet or through WiFi using a router physically mounted on the REMOVI structure.

Table 2 and Table 3 show the information sent by the practitioner’s computer to configure the H1 exoskeleton. Table 4 and Table 5 show the information sent by the same computer to setup the REMOVI PBWS.

The H1 exoskeleton also sends its speed data to the REMOVI PBWS, since the latter needs to adapt its speed to the former’s. These data are sent using the frame described in Table 6 and Table 7.

The H1 exoskeleton sends the data needed to properly monitor and control the session to the therapist’s computer (Table 8 and Table 9). These data are used to compare the actual position of the exoskeleton with the theoretical angles as defined in the programmed gait pattern, and evaluate the force exerted by the patient’s feet.

Finally, the REMOVI PBWS also sends some data to the practitioner’s computer (Table 10 and Table 11). These data are used to monitor and later analyze the master–slave relationship between the two HYBRID subsystems, and the rotating arms’ behaviors.

## 4. Results

HYBRID is a single solution that provides double assistance: (a) an exoskeleton and (b) a stabilization and weight support system. Both devices are independent but work in unison. Each has its own controller and implements a master/slave relationship, with the REMOVI adapting itself to the H1’s pace thanks to the distance measured using the on-board ultrasound sensor of the former.

Therefore, we must ensure that there is a proper coordination between the two subsystems so that the start process of both does not feel disjointed and there is no perceptible delay between them. On top of that, the distance between the H1 and REMOVI should not vary greatly.

To check if these aims were achieved, we measured the delay between several events. We recorded the delay between the instant the patient pressed the button to activate the joint’s movement and the time at which they actually started moving. We also registered how much time passed between the H1 and REMOVI begininning to move. These experiments were performed with three healthy individuals (height: 176 ± 10 cm, age: 25 ± 2 years, weight: 75 ± 21 kg). These participants were unaffiliated with the project and did not participate in any way in the research and design process of the proposed solution. All three were in the standing position with the suspension harness fitted when they activated the exoskeleton in the continuous gait mode, displacing themselves 3 m in a straight line. This experiment was repeated three times, with a support of 30%, 50%, and 70% of their own weight, respectively.

**Figure 15:**
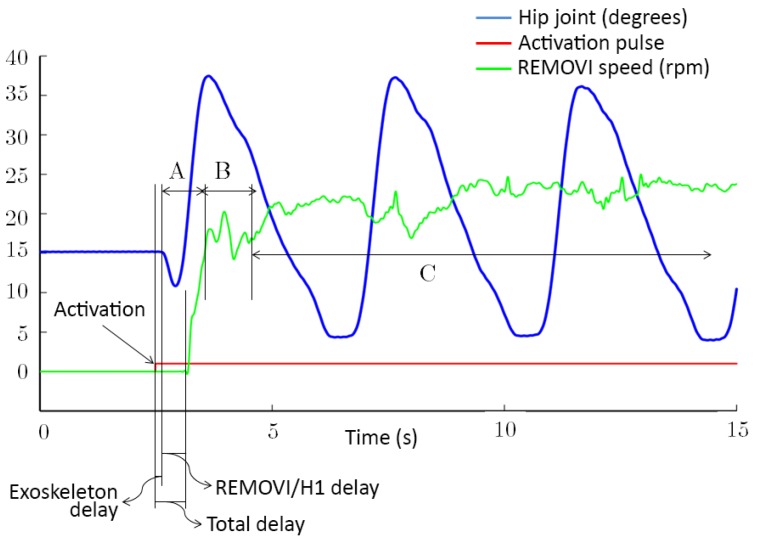
H1–REMOVI synchronization results.

Figure 15 shows the results for the described tests. The mean delay between the button press and the moment the joints started to move was 21 ± 71 ms. The mean delay between this event and the time the REMOVI started to move was 305 ± 287 ms. Therefore, the mean delay between the button press and the instant the user started to walk was 326 ± 293 ms.

## 5. Discussion

### 5.1. H1 and REMOVI Synchronization

After studying the data shown in Figure 15 we identified two transition periods (*A* and *B*) and a permanent regime (*C*) for the REMOVI tractor motors. *A* corresponds to the transition between the exoskeleton start and the beginning of REMOVI’s movement. *B* corresponds to the synchronization process between the H1 and REMOVI speeds. During this process the developed system is using the ultrasounds to match both movements. When *C* is reached, the mean speed of the REMOVI is stable, even if there are some small fluctuations to correct positional drifts. Such drifts are usually a consequence of the user’s movements, posture, and changes in pace due to lengthier strides. However, in some cases the culprits are irregularities in the floor or small wheel slippages. The dynamic range of these fluctuations was quantified as 7 ± 2.58 rpm, while the mean registered speed was 21.2 ± 3 rpm.

Taking these data into account, it can be concluded that the REMOVI properly followed the displacement induced by the H1 without losing stability, even with the added errors due to the terrain and the patient’s movements. The presented values were low enough to be negligible in a rehabilitation environment, with low movement speeds. The users reported that they did not feel a sharp transition and unanimously qualified the starting process as comfortable.

Therefore, our device is also able to provide the promising “repeating without repetition” approach [52], as it permits a variation in the task of gait training. By allowing the patient to wander around the environment, we can avoid the potential loss of adherence due to the boredom that usually arises when walking on a static treadmill. HYBRID also makes the repetitive task more engaging by naturally inducing differences between gait cycles. Such variations within the task have been proven beneficial for the rehabilitation process [53].

### 5.2. Patient’s Position

It is important to discuss an aspect of the development process that ended up being specially relevant: the position of the patient relative to the REMOVI subsystem. The design of HYBRID takes into account the anthropometric characteristics of the target users and several spatial and temporal features like the stride length, cadence, and speed, which together determine the ideal separation between the user and the PBWS. This separation must be maintained while the patient’s center of gravity is kept inside the support base during both the elevation/approach procedure and the actual gait movement. Stability and maneuverability have also been taken into account. A study of the actual comfort experienced by the user resulted in the addition of hand holds to the main structure. After careful study and consideration, it was decided that the user’s position relative to the REMOVI after the elevation must fit steps that advance between 20 and 40 cm at a speed of up to 0.28 m/s (covering the mean walking speed for patients with supervised walkers [54]).

## 6. Conclusions

This article introduces the HYBRID ambulatory trainer—a system capable of inducing a gait movement on the lower limbs while displacing at a proper pace, simultaneously providing partial body weight support and stabilizing the patient during the movement. It also features a combined elevation and approach movement that can easily, quickly, safely, and comfortably transfer the user from the wheelchair to the standing position and vice versa.

This work has taken into account the needs of motor-disabled and low-muscle-tone patients as well as the experience of clinical practitioners. The iterative development of HYBRID involved several partners and produced a two-part system that combines a lower limb 6DoF exoskeleton (H1) with a PBWS moving platform (REMOVI). On top of that, the proposed system is more than a gait trainer; it is capable of acquiring and storing the patient’s data to characterize them. HYBRID is also compatible with external photogrammetry devices and other physiological parameter sensors that can synergize with the abundant captured data and characterize the user.

This system improves on the existing devices present in the literature in several ways. First of all, it combines the gait induction and weight support approaches to rehabilitation, integrating them in an ambulatory platform that can properly stimulate the proprioceptive system, posture, and dynamic balance of the patient. HYBRID also offers a novel semiautonomous elevation and transfer system. Moreover, both the H1 exoskeleton and the REMOVI PBWS maintain the features of the most popular solutions and, in some cases, improve upon them (i.e., powered ankle joints, data logging, consideration of anthropometry, gait patterns obtained using photogrammetry, a graphical user interface for the therapist). More importantly, HYBRID offers a high level of security, comfort, and ease of use—aspects paramount to the real use of a device in a clinical environment, where so often time is of the essence.

Table 12 summarizes the main contributions of this work, which is divided into two subsystems, detailed separately in this article.

Table 13 compares the features of the HYBRID system with those of other gait trainers in the literature. In this table, the column “Interaction” specifies how the exoskeleton and the weight support systems interact with each other. The “Exoskeleton” column indicates if the exoskeleton is fixed to a platform. Finally, the “Transfer” column shows if the device has any kind of wheelchair-to-standing position transference system. From this table, it can be seen that HYBRID provides some features not present in any other published device:

- The exoskeleton is not fixed to an external platform, making a more gradual weight discharge and a greater freedom of movement possible.
- The PBWS subsystem can support up to 100% of the user’s weight.
- The exoskeleton and the weight support system maintain distances using ultrasounds, without needing cables or pressure sensors.
- The system can transfer the patient from a wheelchair to a standing position and vice versa.

## Figures and Tables

**Table 1:** Actuated degrees of freedom (DoF) and range of motion (ROM) of H1 exoskeleton joints. Sign criteria: positive for flexion (dorsiflexion for ankle) and negative for extension (plantarflexion for ankle).

Joint	DOF	Design	ROM
Hip	Flexo/extension	Actuated	100°/−20°
	Addu/abduction	Free	10°/−10°
Knee	Flexo/extension	Actuated	100°/−5°
Ankle	Dorsi/plantarflexion	Actuated	20°/−20°

**Table 2:** H1 configuration frame.

Lock	Gait Mode	Speed

**Table 3:** H1 configuration frame parameters.

Parameter	Value	Description
Lock	0	Forced standing position disabled
	1	Forced standing position enabled
Gait Mode	0	Continuous mode
	1	Step-by-step mode
Speed	0–25	H1 speed (rpm)

**Table 4:** REMOVI configuration frame.

Transfer Mode	Arms Command	User’s Weight	Support	Displacement Mode

**Table 5:** REMOVI configuration frame parameters.

Parameter	Value	Description
Transfer Mode	0	Therapist manually controls elevation
	1	Therapist uses automatic elevation
Arms Command	0	Move arms upwards
	1	Move arms downwards
	2	Stop arms movement
User’s Weight	0–100	User’s weight in kg
Support	0–100	Percentage of support to apply
Displacement Mode	0	Therapist uses the joystick to manually move the system
	1	Therapist uses automatic displacement

**Table 6:** H1 to REMOVI data frame.

Advance	Speed

**Table 7:** H1 to REMOVI data frame parameters.

Parameter	Value	Description
Advance	0	H1 and REMOVI disabled
	1	H1 and REMOVI enabled
Speed	0–3	H1 speed (m/s)

**Table 8:** H1 to PC data frame. FSR: force-sensing resistors.

Right Angles	Right FSR	Right Pattern	Left Angles	Left FSR	Left Pattern

**Table 9:** H1 to PC data frame parameters.

Parameter	Value	Description
Right Angles	−20 to 100/−5 to 100/−20 to 20	Measured angles (degrees) for the right hip/knee/ankle
Right FSR	0 to 5	Measured force (V) by the right FSR
Right Pattern	−20 to 100/−5 to 100/−20 to 20	Theoretical angles (degrees) for the right hip/knee/ankle as determined by the gait pattern
Left Angles	−20 to 100/−5 to 100/−20 to 20	Measured angles (degrees) for the left hip/knee/ankle
Left FSR	0 to 5	Measured force (V) by the left FSR
Left Pattern	−20 to 100/−5 to 100/−20 to 20	Theoretical angles (degrees) for the left hip/knee/ankle as determined by the gait pattern

**Table 10:** REMOVI to PC data frame.

Force X	Force Y	Force Z	Angle	Distance	Speed

**Table 11:** REMOVI to PC data frame parameters.

Parameter	Value	Description
Force X	0–100	Measured force (kg) for the *X* axis
Force Y	0–100	Measured force (kg) for the *Y* axis
Force Z	0–100	Measured force (kg) for the *Z* axis
Angle	0–90	Measured angle (degrees) for the arms’ rotations
Distance	0–100	Measured distance (cm) by ultrasounds between the patient and the REMOVI
Speed	0–3	Measured speed (m/s) for the REMOVI

**Table 12:** HYBRID system’s main contributions.

Subsystem and Contributions	Aim
**H1 Exoskeleton**	
Transmission of movement to patient’s joints
Gait pattern generation	Gait induction
Bilateral coordination between lower limbs and joints	
Gait customization to patient’s needs
Forced standing position in a rigid plane	Forced standing position
**REMOVI PBWS**	
Semiautonomous transfer and elevation from wheelchair to standing position	Partial weight suspension
Partial weight suspension adaptable to load level	
Ease of balancing movements with upper limb support
Increased stability in three planes: sagittal, transversal and frontal	Movement support
Increased security due to fall avoidance	

**Table 13:** Comparison between HYBRID and other gait trainers.

Device	Actuated DoF	PBWS	Support	Interaction	Exoskeleton	Transfer
WalkTrainer	6 DoF pelvis, 3 DoF per leg	Partial	Harness	Potentiometer	Fixed	No
Nature-Gaits	2 DoF pelvis, 3 DoF per leg	Partial	Harness	No	Fixed	No
SUBAR	3 DoF per leg	Only stability	Waist	No	Fixed	No
EXPOS	3 DoF per leg	Only stability	Waist	No	Fixed	No
MLLRE	3 DoF per leg	Partial	Harness	Pressure	Fixed	No
MOPASS	3 DoF per leg	Only stability	Waist	No	Fixed	No
HYBRID	3 DoF per leg	Full	Harness	Ultrasound	Not fixed	Yes

## References

[1] Wirz M., Zemon D.H., Rupp R., Scheel A., Colombo G., Dietz V., Hornby T.G. (2005). Effectiveness of automated locomotor training in patients with chronic incomplete spinal cord injury: A multicenter trial. Arch. Phys. Med. Rehabil..

[2] Hornby T.G., Zemon D.H., Campbell D. (2005). Robotic-assisted, body-weight–supported treadmill training in individuals following motor incomplete spinal cord injury. Phys. Ther..

[3] Husemann B., Müller F., Krewer C., Heller S., Koenig E. (2007). Effects of locomotion training with assistance of a robot-driven gait orthosis in hemiparetic patients after stroke: A randomized controlled pilot study. Stroke.

[4] Lam T., Wolfe D.L., Eng J., Domingo A. (2010). Lower limb rehabilitation following spinal cord injury. Spinal Cord Inj. Rehabil. Evid..

[5] Wolfe D.L., Hsieh J.T., Mehta S. (2010). Rehabilitation practices and associated outcomes following spinal cord injury. Spinal Cord Inj. Rehabil. Evid..

[6] Krakauer J.W. (2006). Motor learning: Its relevance to stroke recovery and neurorehabilitation. Curr. Opin. Neurol..

[7] Reinkensmeyer D.J., Wolbrecht E.T., Chan V., Chou C., Cramer S.C., Bobrow J.E. (2012). Comparison of 3D, assist-as-needed robotic arm/hand movement training provided with Pneu-WREX to conventional table top therapy following chronic stroke. Am. J. Phys. Med. Rehabil..

[8] MacKay-Lyons M. (2002). Central pattern generation of locomotion: A review of the evidence. Phys. Ther..

[9] Kazerooni H., Amundson K., Angold R., Harding N. (2014). Exoskeleton and Method for Controlling a Swing Leg of the Exoskeleton. U.S. Patent.

[10] Kawamoto H., Sankai Y. (2002). Power assist system HAL-3 for gait disorder person. Computers Helping People with Special Needs. ICCHP 2002.

[11] Farris R.J., Quintero H.A., Goldfarb M. (2011). Preliminary evaluation of a powered lower limb orthosis to aid walking in paraplegic individuals. IEEE Trans. Neural Syst. Rehabil. Eng..

[12] Esquenazi A., Talaty M., Packel A., Saulino M. (2012). The ReWalk powered exoskeleton to restore ambulatory function to individuals with thoracic-level motor-complete spinal cord injury. Am. J. Phys. Med. Rehabil..

[13] Sczesny-Kaiser M., Trost R., Aach M., Schildhauer T.A., Schwenkreis P., Tegenthoff M. (2019). A randomized and controlled crossover study investigating the improvement of walking and posture functions in chronic stroke patients using HAL exoskeleton—The HALESTRO study (HAL-Exoskeleton STROke study). Front. Neurosci..

[14] Jansen O., Grasmuecke D., Meindl R.C., Tegenthoff M., Schwenkreis P., Sczesny-Kaiser M., Wessling M., Schildhauer T.A., Fisahn C., Aach M. (2018). Hybrid Assistive Limb exoskeleton HAL in the rehabilitation of chronic spinal cord injury: Proof of concept; the results in 21 patients. World Neurosurg..

[15] Baunsgaard C.B., Nissen U.V., Brust A.K., Frotzler A., Ribeill C., Kalke Y.B., León N., Gómez B., Samuelsson K., Antepohl W. (2018). Gait training after spinal cord injury: Safety, feasibility and gait function following 8 weeks of training with the exoskeletons from Ekso Bionics. Spinal Cord.

[16] Wang S., Wang L., Meijneke C., Van Asseldonk E., Hoellinger T., Cheron G., Ivanenko Y., La Scaleia V., Sylos-Labini F., Molinari M. (2014). Design and control of the MINDWALKER exoskeleton. IEEE Trans. Neural Syst. Rehabil. Eng..

[17] Chen B., Zhong C.H., Zhao X., Ma H., Guan X., Li X., Liang F.Y., Cheng J.C.Y., Qin L., Law S.W. (2017). A wearable exoskeleton suit for motion assistance to paralysed patients. J. Orthop. Transl..

[18] Hyon S.H., Hayashi T., Yagi A., Noda T., Morimoto J. Design of hybrid drive exoskeleton robot XoR2. Proceedings of the 2013 IEEE/RSJ International Conference on Intelligent Robots and Systems.

[19] Barbeau H., Blunt R. (1991). A novel interactive locomotor approach using body weight support to retrain gait in spastic paretic subjects. Plast. Motoneuronal Connect..

[20] Wernig A., Müller S. (1991). Improvement of walking in spinal cord injured persons after treadmill training. Plast. Motoneuronal Connect..

[21] Wernig A., Nanassy A., Müller S. (1998). Maintenance of locomotor abilities following Laufband(treadmill) therapy in para- and tetraplegic persons: Follow-up studies. Spinal Cord.

[22] Colombo G., Jorg M., Dietz V. Driven gait orthosis to do locomotor training of paraplegic patients. Proceedings of the 22nd Annual International Conference of the IEEE Engineering in Medicine and Biology Society (Cat. No. 00CH37143).

[23] Hesse S. (2001). Locomotor therapy in neurorehabilitation. NeuroRehabilitation.

[24] Veneman J.F., Kruidhof R., Hekman E.E., Ekkelenkamp R., Van Asseldonk E.H., Van Der Kooij H. (2007). Design and evaluation of the LOPES exoskeleton robot for interactive gait rehabilitation. IEEE Trans. Neural Syst. Rehabil. Eng..

[25] Banala S.K., Kim S.H., Agrawal S.K., Scholz J.P. Robot assisted gait training with active leg exoskeleton (ALEX). Proceedings of the 2008 2nd IEEE RAS & EMBS International Conference on Biomedical Robotics and Biomechatronics.

[26] Jin X., Cui X., Agrawal S.K. Design of a cable-driven active leg exoskeleton (c-alex) and gait training experiments with human subjects. Proceedings of the 2015 IEEE International Conference on Robotics and Automation (ICRA).

[27] Stauffer Y., Allemand Y., Bouri M., Fournier J., Clavel R., Métrailler P., Brodard R., Reynard F. (2008). The WalkTrainer—A new generation of walking reeducation device combining orthoses and muscle stimulation. IEEE Trans. Neural Syst. Rehabil. Eng..

[28] Luu T.P., Low K.H., Qu X., Lim H.B., Hoon K.H. (2014). Hardware development and locomotion control strategy for an over-ground gait trainer: NaTUre-Gaits. IEEE J. Transl. Eng. Health Med..

[29] Kong K., Tomizuka M., Moon H., Hwang B., Jeon D. (2008). Mechanical design and impedance compensation of SUBAR (Sogang University’s Biomedical Assist Robot). Proceedings of the 2008 IEEE/ASME International Conference on Advanced Intelligent Mechatronics.

[30] Kong K., Jeon D. (2006). Design and control of an exoskeleton for the elderly and patients. IEEE/ASME Trans. Mechatron..

[31] Zhang C., Liu G., Li C., Zhao J., Yu H., Zhu Y. (2016). Development of a lower limb rehabilitation exoskeleton based on real-time gait detection and gait tracking. Adv. Mech. Eng..

[32] Guo Z., Yu H., Yin Y.H. (2014). Developing a mobile lower limb robotic exoskeleton for gait rehabilitation. J. Med. Devices.

[33] Kuzmicheva O., Martinez S.F., Krebs U., Spranger M., Moosburner S., Wagner B., Gräser A. Overground robot based gait rehabilitation system MOPASS-overview and first results from usability testing. Proceedings of the 2016 IEEE International Conference on Robotics and Automation (ICRA).

[34] Canela M., del Ama A.J., Pons J.L. (2013). Design of a pediatric exoskeleton for the rehabilitation of the physical disabilities caused by cerebral palsy. Converging Clinical and Engineering Research on Neurorehabilitation.

[35] Bortole M., Pons J. (2013). Development of a exoskeleton for lower limb rehabilitation. Converging Clinical and Engineering Research on Neurorehabilitation.

[36] Bortole M., Venkatakrishnan A., Zhu F., Moreno J.C., Francisco G.E., Pons J.L., Contreras-Vidal J.L. (2015). The H2 robotic exoskeleton for gait rehabilitation after stroke: Early findings from a clinical study. J. Neuroeng. Rehabil..

[37] De Mauro A., Carrasco E., Oyarzun D., Ardanza A., Frizera-Neto A., Torricelli D., Pons J.L., Agudo A.G., Florez J. (2012). Advanced hybrid technology for neurorehabilitation: The HYPER project. Advances in Robotics and Virtual Reality.

[38] Moreno J., Brunetti F., Pons J. An autonomous control and monitoring system for lower limb orthosis: the gait project case. Proceedings of the 26th Annual International Conference of the IEEE Engineering in Medicine and Biology Society.

[39] Moreno J., Pons J.L., Koutsou A. (2009). The Rehabot-Knee Project Approach for Recovery of Neuromuscular Control of the Knee With Controllable Braces. Int. J. Rehabil. Res..

[40] Del Ama A.J., Moreno J.C., Gil-Agudo A., de-los Reyes A., Pons J.L. (2011). Online assessment of human–robot interaction for hybrid control of walking. Sensors.

[41] Bortole M., Urendes E.J., Pons J.L. (2012). Integración de una plataforma híbrida para rehabilitación y compensación funcional de la marcha. Actas de las XXXIII Jornadas de Automática.

[42] Asín-Prieto G., Urendes E., Gallego J., Moreno J.C., Pons J.L. Monitorización de la estabilidad de la marcha con exoesqueletos basada en información propioceptiva. Proceedings of the CRIA—Congreso Regional en Instrumentación Avanzada (CRIA 2014).

[43] Asín-Prieto G., Moreno J.C. (2014). BioMot Project: Deliverable D4.1 – Physically Based Simulations with Partial Demonstrators I.

[44] Cesarani A., Alpini D. (1999). MCS Organization of the Equilibrium System. Vertigo and Dizziness Rehabilitation.

[45] Kirtley C. (2006). Clinical Gait Analysis: Theory and Practice.

[46] Wiggin M.B., Sawicki G.S., Collins S.H. An exoskeleton using controlled energy storage and release to aid ankle propulsion. Proceedings of the 2011 IEEE International Conference on Rehabilitation Robotics.

[47] Meyer P.F., Oddsson L.I., De Luca C.J. (2004). The role of plantar cutaneous sensation in unperturbed stance. Exp. Brain Res..

[48] Benjumea A.C. (2001). Datos antropométricos de la población laboral española. Prevención, trabajo y salud: Revista del Instituto Nacional de Seguridad e Higiene en el Trabajo.

[49] Winter D.A. (2009). Biomechanics and Motor Control of Human Movement.

[50] Riener R., Lunenburger L., Jezernik S., Anderschitz M., Colombo G., Dietz V. (2005). Patient–cooperative strategies for robot–aided treadmill training: First experimental results. IEEE Trans. Neural Syst. Rehabil. Eng..

[51] Asín-Prieto G., Collantes I., Moreno J.C., Pons J.L. (2012). Diseño de una órtesis motorizada de tobillo para rehabilitación de ictus con un enfoque TOP–DOWN. Actas de las XXXIII Jornadas de Automática.

[52] Cano-de-la-Cuerda R., Molero-Sánchez A., Carratalá-Tejada M., Alguacil-Diego I., Molina-Rueda F., Miangolarra-Page J., Torricelli D. (2015). Theories and control models and motor learning: Clinical applications in neurorehabilitation. Neurología.

[53] Shea C.H., Kohl R.M. (1991). Composition of practice: Influence on the retention of motor skills. Res. Q. Exerc. Sport.

[54] Van Hedel H.J. (2009). Gait speed in relation to categories of functional ambulation after spinal cord injury. Neurorehabil. Neural Repair.

